# Asymptomatic malaria in pregnancy and associated risk factors in Majang Zone, Gambella Region, Southwest Ethiopia: a hard-to-reach malaria hotspot

**DOI:** 10.1186/s12936-024-05041-7

**Published:** 2024-07-15

**Authors:** Aklilu Alemayehu, Ashenafi Abossie, Ahmed Zeynudin, Joseph Beyene, Delenasaw Yewhalaw

**Affiliations:** 1 School of Medical Laboratory Science, Institute of Health, Jimma University, Jimma, Ethiopia; 2 Department of Medical Laboratory Science, College of Medicine and Health Science, Arba Minch University, Arba Minch, Ethiopia; 3 Department of Health Research Methods, Evidence, and Impact, McMaster University, Hamilton, ON Canada; 4 Tropical and Infectious Diseases Research Centre, Jimma University, Jimma, Ethiopia

**Keywords:** Asymptomatic malaria in pregnancy, Anaemia, Prevalence, Risk factors, qPCR, Majang, Gambella

## Abstract

**Background:**

Asymptomatic malaria in pregnancy (AMiP) is a daunting public health problem with multifaceted adverse outcomes for mothers, fetuses, newborns and beyond. This study aimed to assess the prevalence and risk factors of AMiP and anaemia in Majang Zone, Gambella, Southwest Ethiopia.

**Methods:**

A facility-based cross-sectional study was conducted among 425 pregnant women attending the antenatal care (ANC) clinics of five health facilities in the Majang Zone from November 2022 to February 2023. Sociodemographic, obstetric, and anti-malarial intervention data were collected using an interviewer-administered questionnaire. A capillary blood specimen was collected to diagnose malaria and anaemia as well as determine the blood group. Malaria was diagnosed by rapid diagnostic test (RDT), microscopy, and quantitative polymerase chain reaction (qPCR). Statistical analyses were done by Statistical Package for Social Science (SPSS) version 26.0. The association between dependent and independent variables was assessed by multivariable binary logistic regression, considering P < 0.05 statistically significant. The magnitude of associations was quantified with the adjusted odds ratio (AOR) along with the corresponding 95% confidence interval (CI).

**Results:**

The overall prevalence of AMiP was 15.3% (95% CI 12.1, 18.9). It was 11.3% (95% CI 8.4, 14.7) by RDT, 11.8% (95% CI 8.9, 15.2) by microscopy and 17.6% (95% CI 11.7, 24.9) by qPCR. *Plasmodium falciparum*, moderate parasitaemia and submicroscopic infection accounted for 55.4% of the AMiP prevalence, 50.8% of the parasite density, and 41.6% of the qPCR-positive AMiP, respectively. Nearly 32.3% of pregnant women with AMiP carried gametocytes. Risk factors of AMiP were: not utilizing insecticide-treated net (ITN) within the previous week (AOR: 9.43 95% CI 1.57, 56.62), having a history of malaria within the previous year (AOR: 2.26 95% CI 1.16, 4.42), lack of indoor residual spraying (IRS) within the previous year (AOR: 3.00 95% CI 1.50, 6.00), and ANC contact below two rounds (AOR: 4.28 95% CI 2.06, 8.87). The prevalence of anaemia was 27.7% (95% CI 23.6, 32.1), and it was higher among AMiP-positives (56.9%) than the negatives (22.5%) (P: 000).

**Conclusion:**

The prevalence of AMiP and anaemia was high, and remained as a critical public health problem in the study area. Focus on the identified risk factors and introduction of more sensitive diagnostic tools should be considered to mitigate AMiP in the study area.

## Background

Asymptomatic malaria refers to *Plasmodium* infection without apparent symptoms of illness [1]. People living in malaria-endemic areas often have asymptomatic malaria due to partially developed immunity [1–3]. Besides, pregnant women in these settings carry asymptomatic malaria due to parasite sequestration in the placenta [1, 4, 5]. It is often left untreated due to poor treatment-seeking behaviour combined with the low sensitivity of conventional diagnostic tools in these areas [6, 7]. Thus, asymptomatic malaria is a critical problem for pregnant women and the general public [2, 3, 5, 8, 9]. Malaria in pregnancy (MiP) is a major avertible cause of morbidity, mortality, and poor birth outcomes, mainly in sub-Saharan Africa [10–12]. Its adverse results involve mothers, fetuses, newborns, children, and future generations [5, 13, 14]. In high and stable transmission settings, AMiP often leads to maternal anaemia, placental malaria, prematurity, and low birth weight (LBW) [14, 15]. The other adverse effects of MiP are recurrence and the risk of transmission [5, 9, 16, 17]. Consequently, pregnant women might be a human infectious reservoir of malaria due to their high-density parasitaemia, attractiveness to mosquitoes, and drug ineligibility [5, 9, 18]. Generally, an epidemiologic and economic impact of MiP is substantial [3, 5, 14].

An AMiP is a daunting public health problem worldwide [14, 19–21]. A systematic review and meta-analysis (SRMA) report in 2017 showed a 10.8% global prevalence of AMiP [20]. A facility-based cross-sectional study conducted in peri-urban areas of Colombia reported a 5.3% prevalence of MiP (half of which was AMiP) [22]. Community-based studies conducted in Odisha, India, and Chittagong Hill District, Bangladesh, reported a 50.3% [21] and 3.2% [23] prevalence of AMiP, respectively. Moreover, the prevalence and adverse effects of AMiP are marked in sub-Saharan Africa, where a SRMA reported a 26.1% prevalence [19]. Consistently, a high prevalence of AMiP was reported from facility-based cross-sectional studies conducted in Burkina Faso (23.9%) [24], Kenya (12.9%) [25], the Democratic Republic of Congo (29.5%) [26], and Tanzania (36.4%) [27]. An AMiP is also a critical public health problem in Ethiopia, where a recently published SRMA reported a 7.2% prevalence [28]. A lower prevalence was reported in North Shoa (5.7%) [29], Boset (2.74%) [30], and Merti (3.6%) [31] districts. Whereas, a higher prevalence was reported from studies done in Gurage Zone (15.2%) [32], West Guji Zone (24.1%) [33], and Arba Minch (9.7%) [34] in Southern Ethiopia, as well as Jawi District (18.1%) in Northwest Ethiopia [35].

Various factors affect the risk of developing AMiP [19, 28]. Sociodemographic factors, such as rural residence [35] and younger age at pregnancy, raise the risk of AMiP [26]. Obstetric factors such as being primigravida and/or secundigravida [19, 24, 34, 35] and being in the second trimester of pregnancy were found to be risk factors for AMiP [24]. Similarly, a lack and/or poor utilization of anti-malarial interventions were often reported to raise the risk of AMiP. Pregnant women whose homes missed IRS in the previous year had more risk of AMiP than their counterparts [33, 35]. Besides, pregnant women who were not always utilizing ITN had a greater risk of AMiP compared to those who always slept under ITN in the previous week [22, 31, 33]. Given the growing shred of evidence on malaria recurrence, it is conceivable that a history of malaria could increase the risk of AMiP in areas where *Plasmodium falciparum* and *Plasmodium vivax* are co-endemic [17, 28, 31, 33, 36]. These factors raise the risk of AMiP and its deadly outcome, threatening the general public health [22].

To avert the deadly costs of MiP, mitigation strategies include effective case management, vector control, and intermittent preventive treatment in pregnancy (IPTp) [1, 12, 15, 37, 38]. Microscopy and RDT are mainstays for its diagnosis. But, these tools fail to detect placental and submicroscopic malaria, both of which usually remain asymptomatic [4, 6, 39]. Histopathology and PCR are sensitive to detect placental and peripheral MiP, respectively [6, 40]. However, these tools are only used in research due to their high cost and technical complexity [4, 39]. To treat uncomplicated *P. falciparum* MiP in the first trimester, the World Health Organization (WHO) recommends artemether-lumefantrine (AL) or quinine, whereas chloroquine is recommended for uncomplicated non-falciparum MiP [1]. Besides, artemisinin-based combination therapy (ACT) is endorsed as a first-line treatment for uncomplicated MiP in the second and third trimesters. On the other hand, parenteral artesunate followed by AL is used to manage severe MiP, regardless of the *Plasmodium* species [1, 39]. To prevent MiP in moderate to high transmission settings, monthly administration of IPTp with sulfadoxine-pyrimethamine onwards from the second trimester is used [1, 37, 38]. Besides, consistent use of ITN is recommended while ensuring good coverage of IRS [38, 41].

Despite all these efforts, pregnant women remained the most affected population group, who are easily accessible to malaria control programmes, but excluded from some chemotherapeutic interventions [9, 18]. Regardless of the WHO recommendation on mitigation of MiP in endemic areas [37], such efforts are suboptimal in Ethiopia, where IPTp has not yet been endorsed, malaria screening and ITN supply have not been incorporated into the ANC programme [42]. Besides, given to the low coverage of ANC in Gambella Region [43] and the high prevalence of asymptomatic malaria in Abobo Woreda [44], it is plausible that pregnant women might be suffering from a hidden burden of AMiP. Moreover, the national and local health information systems do not show data on AMiP and its adverse outcomes, rendering designing targeted interventions difficult [42]. Such a lack of data might sustain the burden and hinder elimination efforts [9, 11]. Thus, studies tailored by population, place, and time are needed to generate fine-tuned data for designing targeted interventions with efficient operational strategies to reduce the burden. Making anti-malarial intervention inclusive of pregnant women optimizes its yield, and accelerates progress toward elimination [18, 45]. Therefore, this study aimed to assess prevalence and associated risk factors of AMiP and anaemia in Majang Zone, Gambella, Southwest Ethiopia.

## Methods

### Study setting and design

**Fig. 1:**
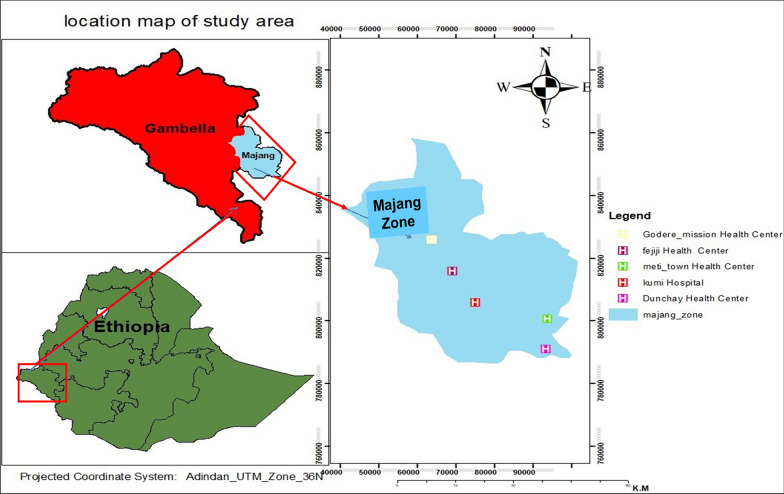
Location map of Majang Zone

A facility-based cross-sectional study was conducted among pregnant women attending ANC clinics at five health facilities in Majang Zone from November 2022, to February 2023. Majang Zone is located in Gambella People's National Regional State of Ethiopia. Metti, 600 km from Addis Ababa and 200 km from Gambella Town, is the capital of the Zone. The Zone is divided into two woredas (Godere and Mengesh Woredas), as well as one administrative town. This Zone has 87,374 population, the majority of whom are rural residents (78%) [46]. An estimated number of reproductive-age women in the Zone is 31,000 [43, 46]. The altitude of Majang Zone ranges from 750 to 1800 m above sea level [46]. The annual rainfall pattern ranges from 1400 to 1800 mm, with seasonal pattern causing seasonal transmission in the Zone, where major and minor transmission occurs from September to December and from April to May, respectively [46, 47]. In addition, the presence of a lake and a dam in the Zone might raise the risk of malaria transmission by providing a breeding site for its vector [47]. Generally, this zone is classified as a malaria-endemic area, where *P. falciparum* and *P. vivax* are co-endemic [42, 47]. In response, the Majang Zone Health Department uses ITN, IRS, and effective case management as pillars of mitigation. The Zone has one hospital, four health centres and about 30 health posts providing preventive and curative services [42]. Totally, the readily available water body, continued deforestation for coffee farm, mobile nature of the population, and poor healthcare access might fuel the burden of AMiP in the area [47] (Fig. 1).

### Sample size and sampling technique

The required sample size was calculated using single population proportion formula by considering a 95% confidence level, 3% margin of error, and 11.2% prevalence of AMiP [35]. After adjusting for a finite population correction and 10% non-response rate, the minimum sample size was estimated to be 422 **(**Eq. 1).

1 $$\begin{gathered} {\text{ n}}_{{\text{o}}} \,{ = }\,\frac{{\left( {{{{\text{Z}}\alpha } \mathord{\left/ {\vphantom {{{\text{Z}}\alpha } 2}} \right. \kern-0pt} 2}} \right)^{{2}} \,{*}\,{\text{P}}\left( {\text{1 - P}} \right)}}{{{\text{d}}^{{2}} }} \hfill \\ {\text{n}}_{{\text{o}}} \,{ = }\,\frac{{\left( {{1}{\text{.96}}} \right)^{{2}} \,{*}\,\,{0}{\text{.112}}\,\left( {{1 - 0}{\text{.112}}} \right)\,}}{{\left( {{0}{\text{.03}}} \right)^{{2}} }}\,{ = }\,{424}{\text{.5224107}}\, \hfill \\ {\text{n}}\,{ = }\,\frac{{{\text{n}}_{{\text{o}}} }}{{{1}\,{ + }\,\left\{ {\frac{{\left( {{\text{n}}_{{\text{o}}} \,{ - }\,{1}} \right)}}{{\text{N}}}} \right\}\,}} \hfill \\ {\text{n}}\,{ = }\,\frac{{{424}{\text{.5224107}}}}{{{1}\,{ + }\,\left\{ {\frac{{\left( {{424}{\text{.5224107}}\,{ - }\,{1}} \right)}}{{3,900}}} \right\}}}\,{ = }\,{382}{\text{.8486118}} \hfill \\ \end{gathered}$$

Formula to calculate sample size for AMiP in Majang Zone. n_o_: initial sample size; N: expected number of pregnant women in the area; n: final sample size; 1-α: confidence level; P: expected proportion; d: margin of error [48].

**Fig. 2:**
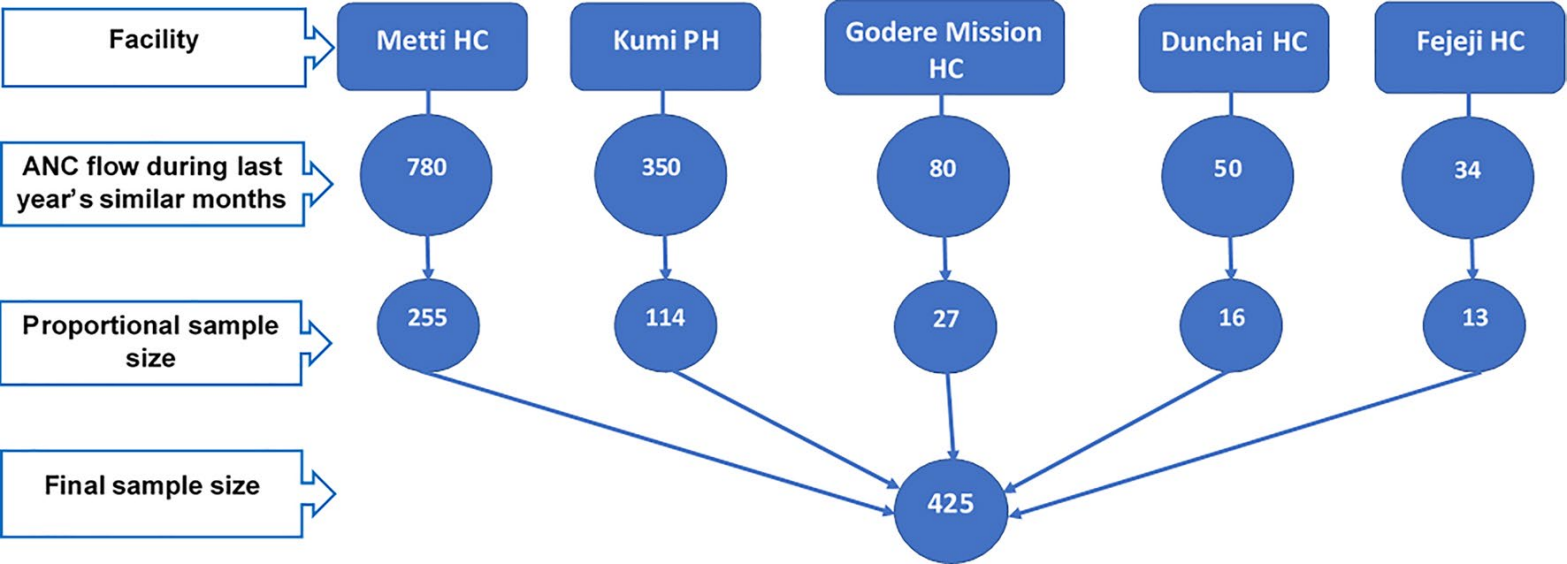
Sampling procedure of this study

A systematic random sampling technique was used to select a representative sample of pregnant women from the ANC logbook by the interval of two. The sample size was proportionally allocated for the five health facilities, based on the flow of ANC clients in similar four months of the previous year. Finally, 425 pregnant women were included in the study (Fig. 2).

### Inclusion and exclusion criteria

Pregnant women who lived in the zone for at least six months and were free of malaria symptoms attending ANC clinics at the five health facilities during the study period were included. However, pregnant women with axial body temperature ≥ 37.5 °C were excluded. Moreover, those who took anti-malarial drugs, took anti-pain drugs, has history of fever, and has a history of malaria within 14 days, 24 h, 48 h, and 28 days prior to the data collection date, respectively were excluded.

### Study variables

*Dependent variable* Asymptomatic malaria in pregnancy.

*Independent variables* Age, marital status, education, occupation, family size, residence, gestational age, parity, gravidity, ITN, IRS, number of ANC contact, blood group, and history of malaria.

### Sociodemographic data collection

Data on sociodemographic, obstetric, anti-malarial intervention, and ANC service utilization of the pregnant women were collected using a pre-tested, structured, interviewer-administered questionnaire that was adapted from the 2016 Ethiopian Demographic Health Survey and the 2015 Malaria Indicator Survey documents. The interviews were conducted in the Amharic language by two trained midwives, and the response of each pregnant woman was recorded on the questionnaire per the instructions.

### Blood specimen collection

Using a disposable pipette, approximately 375 µl capillary blood was collected from a cleaned ring finger of a pregnant woman by pricking it with a sterile lancet. The blood was used to perform malaria RDT (5 µl) and microscopy (8 µl), and prepare dried blood spots (DBSs) (4 circles of 50 µl) on filter paper for qPCR analysis. In addition, the blood was used to determine the haemoglobin (Hb) concentration (10 µl) and blood group (3 drops of 50 µl). The DBSs were set to airdry overnight and packed in a plastic bag containing desiccant. Then, the DBSs were stored at -20 °C at MHC and transported to Tropical and Infectious Diseases Research Centre (TIDRC) of Jimma University, Sekoru for qPCR analysis. The laboratory tests are briefly described as follows.

### Malaria rapid diagnostic test

Onsite diagnosis of *Plasmodium* infection was performed using Bioline™ Malaria Ag *P.f*/Pan RDT (catalog number: 05FK60, Abbott Diagnostics, Republic of Korea) following the manufacturer’s instructions. This malaria RDT is a rapid, qualitative, and three-band differential lateral-flow immunochromatographic Ag detection cassette test that detects HRP2 Ag of *P. falciparum* and pLDH Ag of the four common human malaria-causing *Plasmodium* species in whole blood [49, 50]. The principle of this RDT method is based on the capture of a labelled antibody (LAb) to produce a visible band on a nitrocellulose strip enclosed in a cassette. Following addition of whole blood (1 drop or 5 µl) and assay diluent (4 drops), LAb binds to parasite Ag to form an Ag-Ab complex, which then migrates across the strip and gets captured by a bound antibody (BAb) at test region (T-region), forming a visible band in the result window. The remaining free LAb continues migrating and gets captured by a BAb at control region (C-region) to serve as a quality control. In the presence of band at C-region, no any band, band at *P. falciparum* T-region only, band at Pan T-region only, and band at *P. falciparum* and Pan T-regions is interpreted as negative, *P. falciparum* mono, other species of *Plasmodium*, and *P. falciparum* and other species mixed infection, respectively [50, 51].

### Malaria microscopy

For each participant, thick (6 µl) and thin (2 µl) blood films were prepared on two glass slides. After fixing the thin film in Absolute Methanol for 10–15 s, the films were stained with 3% Giemsa stain for 30–40 min and examined under 1000X magnification using an Olympus CX23 light microscope (Olympus, Japan) by a trained Medical Laboratory Technologist at MHC. The examination involved assessing smears for the presence of *Plasmodium* parasite, identifying species, and determining stages and density. Sexual and asexual parasite densities were determined by counting parasites against 500 and 200 White blood cells (WBCs), respectively. The obtained number was converted into number of parasites/µl, assuming 8.0 × 10^3^ WBCs/µl of blood. A blood film was declared negative if no parasite was observed after examining 200 oil immersion fields [8, 52–54].

### *Plasmodium* species detection and identification by qPCR assay

The molecular detection and identification of the *Plasmodium* species involved genomic deoxyribonucleic acid (DNA) extraction from DBSs by Chelex-100 method and target amplification by qPCR in the molecular laboratory at TIDRC of Jimma University, Sekoru. The extraction involved cutting and placing approximately 3 mm diameter DBSs pieces into 1.5 ml Eppendorf tubes and adding 5% Chelex-100 alkaline solution that was incubated overnight, followed by a series of centrifugation and thermal vortexing steps, resulting in the release of the parasite DNA and the transfer of 0.5 ml of pure DNA into a new Eppendorf tube for storage at −20 °C until the PCR was run.

PCR was used to amplify the species-specific 18S rRNA gene in the extracted DNA via multiplex qPCR using a QuantStudio™ 3 System instrument (Applied Biosystems Incorporated). The process involved the use of pairs of primers and probes specifically designed for simultaneous detection and identification of both *P. falciparum* and *P. vivax*. For *P. falciparum*, F-F (forward primer sequence): TAT TGC TTT TGA GAG GTT TTG TTA CTT TG; F-R (reverse primer sequence): ACC TCT GAC ATC TGA ATA CGA ATGC; and probe sequence: *Pf*-fam (MGB): ACG GGT AGT CAT GAT TGA GTT were used [55]. For *P. vivax*, Pv-1 (forward primer sequence): CGC TTC TAG CTT AAT CCA CAT AAC TG; Pv-2 (reverse primer sequence): AAT TTA CTC AAA GTA ACA AGG ACT TCC AAG; and the probe sequence: Pv-probe (NED-MGB): CGC ATT TTG CTA TTA TGT were used [56]. PCR amplification was conducted on a total volume of 12 µl in a 96-well PCR plate. The PCR mixture was prepared by adding 6 µl (× 2) of PerfeCTa master mix [PerfeCTa^®^ qPCR ToughMix^®^ Low ROX^™^, Lot number: 66181991 (Quantabio)], 0.5 µl (× 2) of each of the *Pf*-Fam and *Pv*-NED probes, 0.4 µl (× 4) of each of the forward and reverse primers, 2 µl of template DNA and 1.4 µl of PCR grade water into each well. The thermal cycling condition involved a hold stage at 50 °C for 2 min and initial denaturation at 95 °C for 2 min, followed by 45 cycles of amplification stage at 95 °C for 3 s and 60 °C for 30 s. PCR-grade water and *P. falciparum* DNA (MR4 BEI) were used as positive and negative controls, respectively. The total run-time of this qPCR process was roughly 65 min [44].

### Haemoglobin concentration and blood group determination

The Hb concentration was determined using a HemoCue^®^ Hb 301 Analyzer (HemoCue^®^ Hb 301 Analyzer AB/Kuvettgatan, Ängelholm, Sweden). About 10 µl whole blood was drawn into the cavity of a microcuvette by capillary action, followed by swift insertion of the microcuvette into the analyzer, which measures absorbance and displays the Hb concentration in g/dl within 10–20 s [57]. Besides, the ABO blood group and Rho type were determined by the haemagglutination test method on slides using ForSure monoclonal IgM antiserums (Setia Scientific Solution, Selangor, Malaysia). This method involved the addition of one drop of each antiserum (anti-A, anti-B, and anti-D) on a clean glass slide (while avoiding mix-up) and addition of one drop of whole blood (about 50 µl) onto each of these antiserums, followed by thorough mixing and visually reading the result (presence or absence of haemagglutination) within two minutes [57].

### Data quality control and statistical analysis

Before data collection, training on collecting blood specimen, running tests, and filling the questionnaire was given by the principal investigator to the data collectors. The questionnaire was pretested in Gambella General Hospital. The principal investigator supervised the data collection process. All laboratory tests were performed strictly following standard operating procedures, job aids, and manufacturers’ instructions. Controls were run before reporting results. Two trained laboratory technologists independently examined the blood films. Whenever there is discrepancy in microscopy results, it was resolved by the third reader (senior laboratory technologist). The collected data were appropriately cleaned, coded, and entered into Epidata version 3.1 and exported into SPSS Version 26 Software for Windows (Chicago, IL, USA). After checking for completeness and consistency, statistical analyses were run. Descriptive and binary logistic regression analyses were conducted to summarize the data and identify the risk factors, respectively. In univariate binary logistic regression analysis, factors with P < 0.2 were taken as candidates for multivariate binary logistic regression analysis, from which those with P < 0.05 were considered statistically significant. An AORs were obtained along with the corresponding 95% CI. The results were illustrated in texts, tables, and figures.

### Operational definitions

- *AMiP* Laboratory confirmed parasitaemia (either RDT or microscopy or qPCR) in the pregnant woman with axial body temperature < 37.5 °C, and no fever in the last 48 h [1].
- *Low parasitaemia*  < 1000 asexual stage parasites/µl [53, 54].
- *Moderate parasitaemia* 1000–9999 asexual stage parasites/µl [53, 54].
- *High parasitaemia*  ≥ 10,000 asexual stage parasites/µl [53, 54].
- *Anaemia* [Hb] < 11.0 g/dl [58].
- *Mild anaemia* [Hb] 10.0–10.9 g/dl [58].
- *Moderate anaemia* [Hb] 7.0–9.9 g/dl [58].
- *Severe anaemia* [Hb] < 7.0 g/dl [58].
- First trimester  ≤ 12 weeks [37].
- *Second trimester* 13–26 weeks [37].
- *Third trimester*  ≥ 27 weeks [37].

## Results

### Sociodemographic, obstetric, and intervention characteristics of the study participants

Totally, 425 pregnant women participated in this study. The mean age of the participants was 26.5 years, with a minimum and maximum of 18 and 42 years, respectively. Majority of them were currently in a married state (84.2%), completed secondary education (35.8%), housewives (63.8%), and rural residents (56.0%). The average number of people per household was 3.2, with a minimum and maximum of one and nine people, respectively. The mean gestational age of the study participants was 22.5 weeks, with a minimum and maximum of six and 41 weeks, respectively. Majority of them were in the second trimester (52.3%), multigravida (46.4%), multipara (43.1%), and blood group O (42.1%). The average number of ANC contacts was 2.02, with a minimum and maximum of one and seven contacts, respectively. The mean Hb concentration was 11.9 g/dl, that ranged from 5.4 g/dl to 20.1 g/dl. Majority of the participants owned at least one ITN (69.9%) and had their home wall sprayed with IRS within the previous year (56.7%). Under one-third of the participants used ITN throughout the previous week (29.6%) and the previous year (30.6%). Over one-third of them had never used ITN within the previous week (34.8%) and within the previous year (33.4%), as well as had a history of malaria within the previous year (40.7%) (Table 1).

**Table 1:** Sociodemographic, obstetric, and malaria intervention characteristics of pregnant women in Majang Zone, Gambella, Southwest Ethiopia, November 2022–February 2023 (n = 425)

Sociodemographic characteristics	n (%)	Obstetric characteristics	n (%)	Intervention characteristics	n (%)
Age		Trimester		ITN availability	
< 20	62 (14.6)	1st Trimester	61 (14.4)	No	128 (30.1)
21–25	131 (30.8)	2nd Trimester	226 (53.2)	Yes	297 (69.9)
26–30	139 (32.7)	3rd Trimester	138 (32.5)	ITN quantity	
> 31	93 (21.9)	Gravidity		0	128 (30.1)
Educational status		Primigravida	162 (38.1)	1	223 (52.4)
Unable to read and write	111 (26.1)	Secundigravida	66 (15.5)	> 2	74 (17.5)
Primary	119 (28.0)	Multigravida	197 (46.4)	ITN using season within the previous year	
Secondary	152 (35.8)	Parity		Not at all	142 (33.4)
College and above	43 (10.1)	Nullipara	170 (40.0)	Outbreak only	18 (4.3)
Marital status		Primipara	183 (43.1)	Rainy season only	135 (31.8)
Never married	23 (5.4)	Multipara	183 (43.1)	All year round	130 (30.6)
Married	358 (84.2)	Number of ANC contact		ITN used in the previous night	
Divorced	26 (6.1)	First	205 (48.2)	No	219 (51.5)
Widowed	18 (4.2)	At least two	220 (51.8)	Yes	206 (48.5)
Occupation		Blood group		Number of days slept under ITN within the previous week	
Student	38 (8.9)	AB	31 (7.3)	Not at all	148 (34.8)
Housewife	271 (63.8)	A	127 (29.9)	1–4	67 (15.8)
Employed	116 (27.3)	B	88 (20.7)	5–6	84 (19.8)
Residence		O	179 (42.1)	7	126 (29.6)
Rural	238 (56.0)	Anemia		IRS sprayed in a home within the previous year	
Urban	187 (44.0)	Severe	9 (2.1)	No	184 (43.3)
Family size		Moderate	47 (11.1)	Yes	241 (56.7)
> 6	33 (7.8)	Mild	62 (14.6)	History of malaria within the previous year	
3–5	213 (50.1)	Negative	307 (72.2)	No	252 (59.3)
1–2	179 (42.1)			Yes	173 (40.7)

### Prevalence and parasite density of asymptomatic malaria in pregnancy

**Fig. 3:**
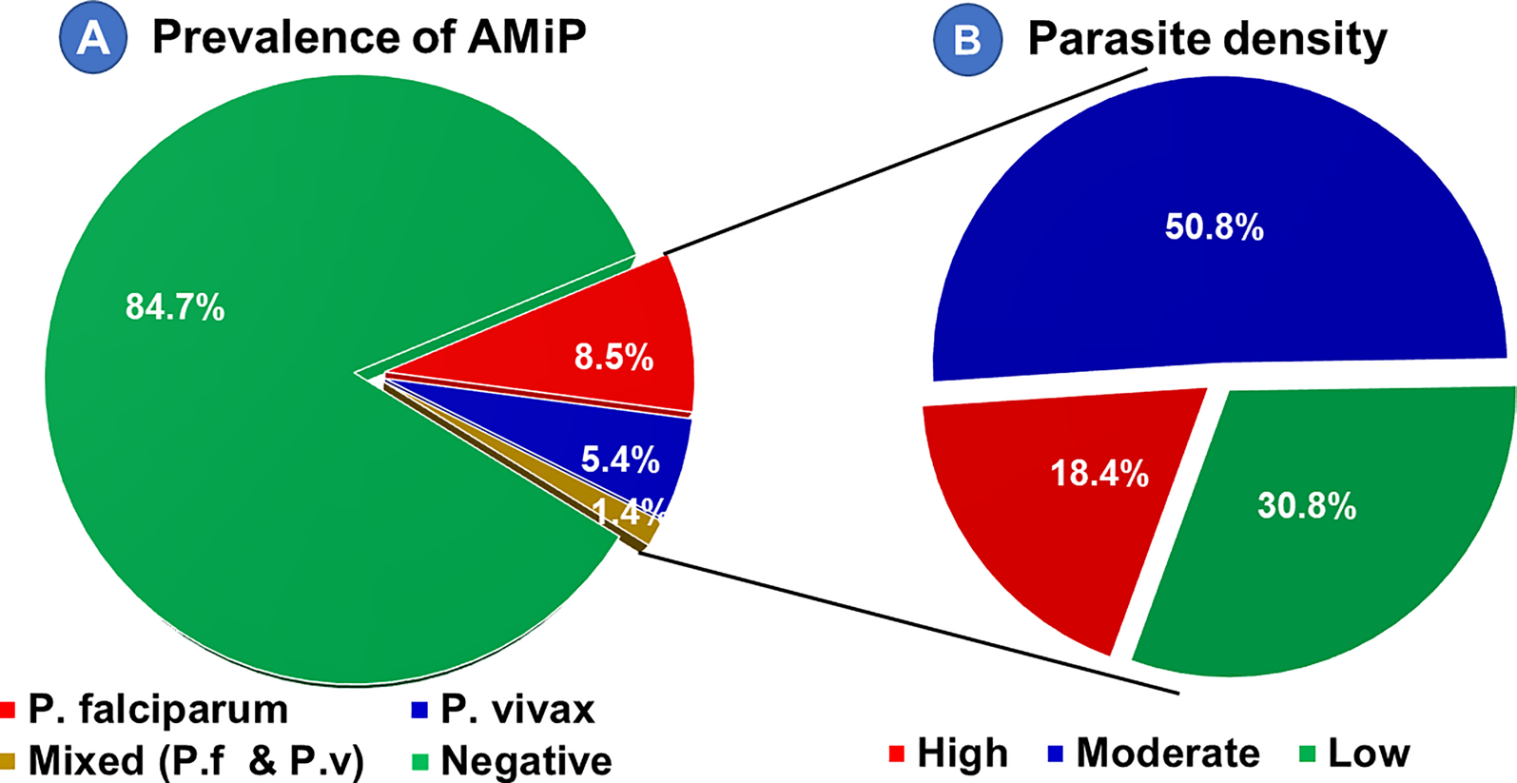
Prevalence of AMiP (**A**) and asexual stage parasite density (**B**) in Majang Zone, Gambella, Southwest Ethiopia, November 2022– February 2023 [(n for prevalence = 65), (n for parasite density = 50)

The overall prevalence of AMiP among pregnant women attending ANC clinics at health facilities in Majang Zone was 15.3% (95% CI 12.0, 19.1). The prevalence was 11.3% (95% CI 8.4, 14.7) by RDT, 11.8% (95% CI 8.9, 15.2) by microscopy and 17.6% (95% CI 11.7, 24.9) by qPCR (P: 0.01). The prevalence of *P. falciparum*, *P. vivax* and mixed (*P. falciparum* and *P. vivax*) were 8.5% (36/425), 5.4% (23/425) and 1.4% (6/425), respectively. Based on parasite counting from thick blood film, the asexual stage parasite density ranged from 64/ µl to 38,400/ µl, with a geometric mean density of 2,683/ µl. High parasitaemia accounted for 18.4% (9/50) of the overall microscopically detected parasitaemia. Besides, about one-third (32.3%) of AMiP cases carried gametocyte stage, making its overall prevalence 4.9% (21/425). About 61.9% (13/21), 28.6% (6/21), and 9.5% (2/21) of these gametocytes belong to *P. vivax*, *P. falciparum,* and mixed species, respectively. Sexual stage parasite density ranged from 64/ µl to 7,560/ µl, with a geometric mean density of 1,213.6/ µl (Fig. 3).

### Distribution of asymptomatic malaria in pregnancy by participants’ characteristics

The prevalence of AMiP was greater among participants who were currently married (86.2%), housewives (67.7%), and rural residents (63.1%) than their corresponding counterparts. Similarly, the prevalence of AMiP was greater among those who did not sleep under ITN during the previous night (76.9%), never used ITN within the previous week (64.6%), never utilized ITN within the previous year (52.3%), had not gotten their home sprayed with IRS within the previous year (75.4%), and had a history of malaria within the previous year (70.8%). The prevalence of AMiP was greater among participants who were in their 2nd trimester (44.6%), primigravida (44.6%), nullipara (47.8%), had only one ANC contact (80.0%) and had blood group O (43.1%) than their comparators (Table 2).

**Table 2:** Species-level prevalence of AMiP by participants’ characteristics in Majang Zone, Gambella, Southwest Ethiopia, November 2022–February 2023 (*n* = 425)

Variable	AMiP				Tested
	*P. f*	*P. v*	Mixed	Overall	
	n (%)	n (%)	n (%)	n (%)	
Prevalence by species	36 (8.5)	23 (5.4)	6 (1.4)	65 (15.3)	425
Age					
< 20	8 (22.2)	7 (30.4)	0 (0)	15 (23.1)	62
21–25	13 (36.1)	7 (30.4)	2 (33.3)	22 (33.8)	131
26–30	7 (19.4)	6 (26.1)	2 (33.3)	15 (23.1)	139
> 31	8 (22.2)	3 (13.0)	2 (33.3)	13 (20.0)	93
Educational status					
Unable to read and write	13 (36.1)	7 (30.4)	4 (66.7)	24 (36.9)	111
Primary	10 (27.8)	1 (4.3)	0 (0)	11 (16.9)	119
Secondary	12 (33.3)	11 (47.8)	2 (33.3)	25 (38.5)	152
College and above	1 (2.8)	4 (17.4)	0 (0)	5 (7.7)	43
Marital status					
Never married	2 (5.6)	1 (4.3)	0 (0)	3 (4.6)	23
Divorced	1 (2.8)	1 (4.3)	1 (16.7)	3 (4.6)	26
Widowed	0 (0)	3 (13.0)	0 (0)	3 (4.6)	18
Married	33 (91.7)	18 (78.3)	5 (84.3)	56 (86.2)	358
Occupation					
Student	4 (11.1)	3 (13.0)	0 (0)	7 (10.8)	38
Housewife	28 (77.8)	11 (47.8)	5 (83.3)	44 (67.7)	271
Employed	4 (11.1)	9 (39.1)	1 (16.7)	14 (21.5)	116
Residence					
Rural	21 (58.3)	15 (65.2)	5 (83.3)	41 (63.1)	238
Urban	15 (41.7)	8 (34.8)	1 (16.7)	24 (36.9)	187
Family size					
1–2	5 (13.9)	2 (8.7)	2 (33.3)	9 (13.8)	179
3–5	17 (47.2)	11 (47.8)	3 (50.0)	31 (47.7)	213
> 6	14 (38.9)	10 (43.5)	1 (16.7)	25 (38.5)	33
ITN availability					
No	12 (33.3)	10 (43.5)	2 (33.3)	24 (36.9)	128
Yes	24 (66.7)	13 (56.5)	4 (66.7)	41 (63.1)	297
ITN quantity					
0	12 (33.3)	10 (43.5)	2 (8.3)	24 (36.9)	128
1	18 (50.0	10 (43.5)	4 (50.0)	32 (49.3)	223
> 2	6 (16.7)	3 (13.0)	0 (0)	9 (13.8)	74
ITN utilization periodicity within the previous year					
Not at all	19 (52.8)	11 (47.8)	4 (66.7)	34 (52.3)	142
During outbreak only	4 (11.1)	3 (13.0)	1 (16.7)	8 (12.2)	18
During rainy season only	9 (25.0)	5 (21.7)	0 (0)	14 (21.5)	135
All year round	4 (11.1)	4 (17.4)	1 (11.1)	9 (13.8)	130
ITN utilization in the previous night					
No	25 (69.4)	19 (82.6)	6 (100)	50 (76.9)	219
Yes	11 (30.6)	4 (17.4)	0 (0)	15 (23.1)	206
Number of nights slept under ITN within the previous week					
Not at all	19 (52.8)	19 (82.6)	4 (66.7)	42 (64.6)	148
1–4	9 (25.0)	2 (8.7)	0 (0)	11 (16.9)	67
5–6	3 (8.3)	1 (4.3)	2 (33.3)	6 (9.2)	84
7	5 (13.9)	1 (4.3)	0 (0)	6 (9.2)	126
IRS sprayed in a home wall within the previous year					
No	25 (69.4)	20 (87.0)	4 (66.7)	49 (75.4)	184
Yes	11 (30.6)	3 (13.0)	2 (33.3)	16 (24.6)	241
History of malaria within the previous year					
No	16 (44.4)	2 (8.7)	1 (16.7)	19 (29.2)	252
Yes	20 (55.5)	21 (91.3)	5 (83.3)	46 (70.8)	173
Trimester					
1st Trimester	6 (16.7)	2 (8.7)	0 (0)	8 (12.3)	61
2nd Trimester	19 (52.8)	12 (52.2)	5 (83.3)	36 (55.4)	226
3rd Trimester	11 (30.6)	9 (39.1)	1 (16.7)	21 (32.3)	138
Gravidity					
Primigravida	16 (44.4)	11 (47.8)	2 (33.3)	29 (44.6)	162
Secundigravida	5 (13.9)	3 (13.0)	2 (33.3)	10 (15.4)	66
Multigravida	15 (41.7)	9 (39.1)	2 (33.3)	26 (40.0)	197
Parity					
Nullipara	17 (47.2)	11 (47.8)	2 (33.3)	30 (46.2)	170
Primipara	5 (13.9)	3 (13.0)	2 (33.3)	10 (15.5)	72
Multipara	14 (38.9)	9 (39.1)	2 (33.3)	25 (38.3)	183
Number of ANC contact					
1	28 (77.8)	19 (82.6)	5 (83.3)	52 (80.0)	205
> 2	8 (22.2)	4 (17.4)	1 (16.72)	13 (20.0)	220
Blood group					
AB	2 (5.6)	2 (8.7)	0 (0)	4 (6.2)	32
A	8 (22.2)	7 (30.4)	2 (33.3)	17 (26.2)	128
B	11 (30.6)	4 (17.4)	1 (16.7)	16 (24.6)	88
O	15 (41.7)	10 (43.5)	3 (50.0)	28 (43.1)	177

### Risk factors of asymptomatic malaria in pregnancy

Sociodemographic, obstetric, anti-malarial intervention, and other characteristics of participants were initially analysed using univariate logistic regression for possible association with AMiP. Nine variables met the inclusion criteria (P < 0.2) to be taken as candidates for multivariable binary logistic regression analysis, where only four variables were found to be statistically significantly associated with AMiP. These were: not utilizing ITN within the previous week [AOR: 9.43 95% CI 1.57, 56.62 (P: 0.014)], having a history of malaria within the previous year [AOR: 2.26 95% CI 1.16, 4.42 (P: 0.016)], lack of IRS spraying within the previous year [AOR: 3.00 95% CI 1.50, 6.00 (P: 0.002)], and ANC contact below two rounds [AOR: 4.28 95% CI 2.06, 8.87 (P: 0.000)] (Table 3).

**Table 3:** Univariate and multivariable binary logistic regression analyses to identify risk factors of AMiP in Majang Zone, Gambella, Southwest Ethiopia, November 2022–February 2023 (*n* = 425)

Variable	AMiP		Total	COR (95% CI)	*p*-value	AOR (95% CI)	*p*-value
	No	Yes					
	*n* (%)	*n* (%)					
Age							
< 20	47 (75.8)	15 (24.2)	62	1.96 (0.86–4.45)	0.109*	2.12 (0.73–6.17)	0.168
21–25	109 (83.2)	22 (16.8)	131	1.24 (0.59–2.61)	0.568	1.10 (0.44–2.76)	0.830
26–30	124 (89.2)	15 (10.8)	139	0.74 (0.33–1.65)	0.466	0.60 (0.22–1.60)	0.308
> 31	80 (86.0)	13 (14.0)	93	1		1	
Educational status							
Unable to read and write	87 (78.4)	24 (21.6)	111	2.01 (0.74–5.91)	0.161*	1.63 (0.44–5.94)	0.461
Primary	108 (90.8)	11 (9.2)	119	0.77 (0.25–2.37)	0.654	0.47 (0.11–1.91)	0.291
Secondary	127 (83.6)	25 (16.4)	152	1.49 (0.53–4.17)	0.442	1.36 (0.38–4.81)	0.637
College and above	38 (88.4)	5 (11.6)	43	1		1	
Marital status							
Single	58 (86.6)	9 (13.4)	67	0.84 (0.39–1.78)	0.645		
Married	302 (84.4)	56 (15.6)	358	1			
Occupation							
Student	31 (81.6)	7 (18.4)	38	1.64 (0.61–4.44)	0.325		
Housewife	227 (83.8)	44 (16.2)	271	1.42 (0.74–2.69)	0.294		
Employed	102 (87.9)	14 (12.1)	116	1			
Residence							
Rural	197 (82.8)	41 (17.2)	238	1.41 (0.82–2.44)	0.213		
Urban	163 (87.2)	24 (12.8)	187	1			
Family size							
1–2	145 (86.6)	25 (14.0)	179	1		1	
3–5	182 (85.4)	31 (14.6)	213	1.05 (0.59–1.85)	0.868	1.66 (0.80–3.47)	0.176
> 6	24 (72.7)	9 (27.3)	33	2.31 (0.96–5.54)	0.061*	2.76 (0.95–8.00)	0.062
ITN availability							
No	104 (81.2)	24 (18.8)	128	1.44 (0.83–2.50)	0.210		
Yes	256 (86.2)	41 (13.8)	297	1			
ITN quantity							
0	104 (81.2)	24 (18.8)	128	1.66 (0.73–3.81)	0.226		
1	191 (85.7)	32 (14.3)	223	1.21 (0.55–2.67)	0.637		
> 2	65 (87.8)	9 (12.2)	74	1			
ITN utilizing season/period within the previous year							
Not at all	108 (76.1)	34 (23.9)	142	4.23 (1.94–9.22)	0.000*	0.43 (0.09–1.99)	0.281
During outbreak only	10 (55.6)	8 (44.4)	18	10.75 (3.40–33.97)	0.000*	2.58 (0.46–12.16)	0.232
During rainy season only	121 (89.6)	14 (10.4)	135	1.56 (0.65–3.73)	0.322	1.01 (0.33–3.05)	0.980
All year round	121 (93.1)	9 (6.9)	130	1		1	
ITN utilization within the previous night							
No	169 (77.2)	50 (22.8)	219	3.77 (2.04–6.96)	0.000*	0.90 (0.30–2.64)	0.849
Yes	191 (92.7)	15 (7.3)	206	1		1	
Number of days slept under ITN within the previous week							
Not at all	106 (71.6)	42 (28.4)	148	7.92 (3.24–19.38)	0.000*	9.43 (1.57–56.62)	0.014**
1–4	56 (83.6)	11 (16.4)	67	3.93 (1.38–11.16)	0.010*	2.45 (0.58–10.32)	0.223
5–6	78 (92.9)	6 (7.1)	84	1.54 (0.47–4.94)	0.469	1.17 (0.29–4.70)	0.827
7	120 (95.2)	6 (4.8)	126	1		1	
IRS sprayed in a home within the within the previous year							
No	135 (73.4)	49 (26.6)	184	5.10 (2.79–9.33)	0.000*	3.00 (1.50–6.00)	0.002**
Yes	225 (93.4)	16 (6.6)	241	1		1	
History of malaria within the within the previous year							
No	233 (92.5)	19 (7.5)	252	4.44 (2.49–7.90)	0.000*	2.26 (1.16–4.42)	0.016**
Yes	127 (73.4)	46 (26.6)	173	1		1	
Gestational age (Trimester)							
1st Trimester	53 (86.9)	8 (13.1)	61	0.84 (0.35–2.02)	0.699		
2nd Trimester	190 (84.1)	36 (15.9)	226	1.05 (0.59–1.89)	0.856		
3rd Trimester	117 (84.8)	21 (15.2)	138	1			
Gravidity							
Primigravida	133 (82.1)	29 (17.9)	162	1.43 (0.80–2.55)	0.220		
Secundigravida	56 (84.8)	10 (15.2)	66	1.17 (0.54–2.58)	0.690		
Multigravida	171 (86.8)	26 (13.2)	197	1			
Parity							
Nullipara	140 (82.4)	30 (17.6)	170	1.35 (0.76–2.41)	0.303		
Primipara	62 (86.1)	10 (13.9)	72	1.02 (0.46–2.24)	0.962		
Multipara	158 (86.3)	25 (13.7)	183	1			
Number of ANC contact							
1	153 (74.6)	52 (25.4)	205	5.41 (2.84–10.29)	0.000*	4.28 (2.06–8.87)	0.000**
> 2	207 (94.1)	13 (5.9)	220	1		1	
Blood group							
AB	28 (87.5)	4 (12.5)	32	0.76 (0.24–2.33)	0.632		
A	111 (86.7)	17 (13.3)	128	0.81 (0.42–1.56)	0.538		
B	72 (81.8)	16 (18.2)	88	1.18 (0.60–2.32)	0.627		
O	149 (84.2)	28 (15.8)	177	1			

*Candidate for multivariable binary logistic regression analysis

**Statistically significant

### Prevalence of anaemia and rate of blood group distribution among pregnant women

**Fig. 4:**
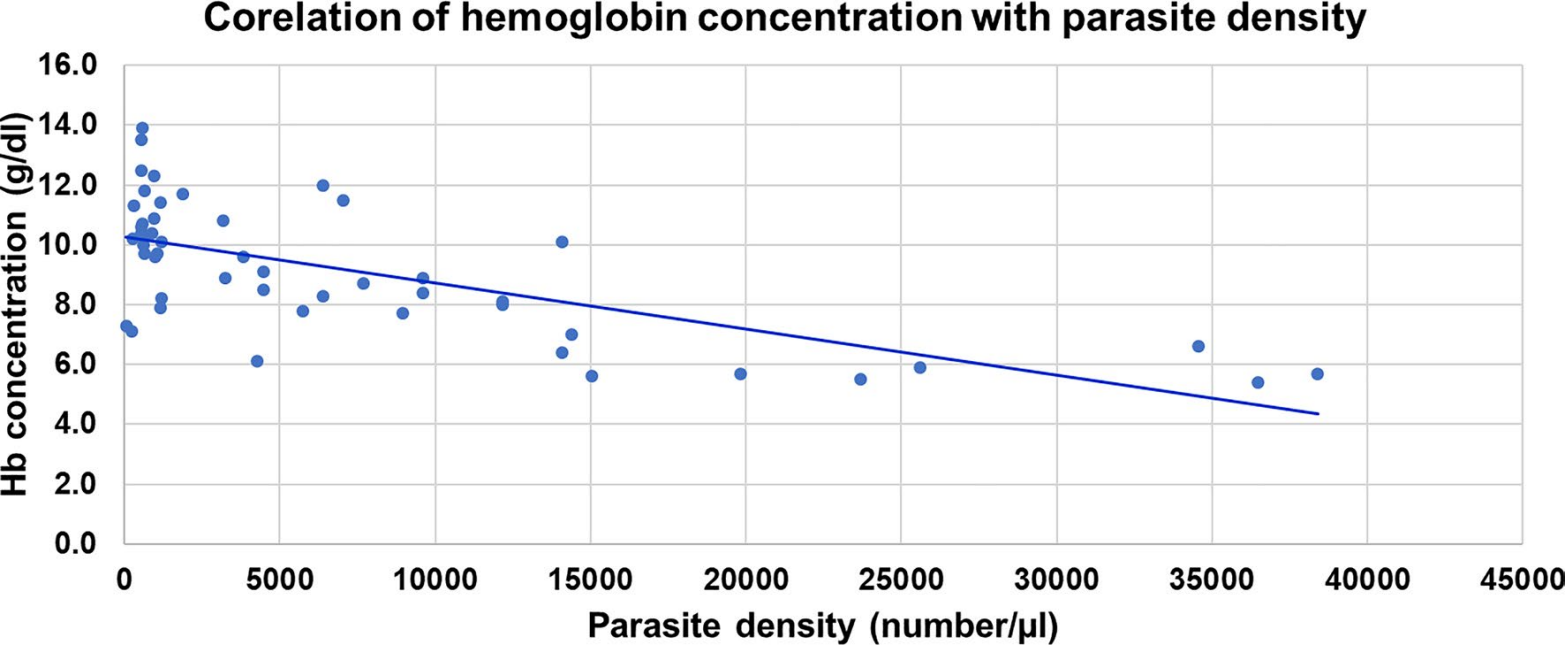
Correlation of haemoglobin concentration with *Plasmodium* parasite density among pregnant women in Majang Zone, Gambella, Southwest Ethiopia, November 2022–February 2023, (n = 50)

**Fig. 5:**
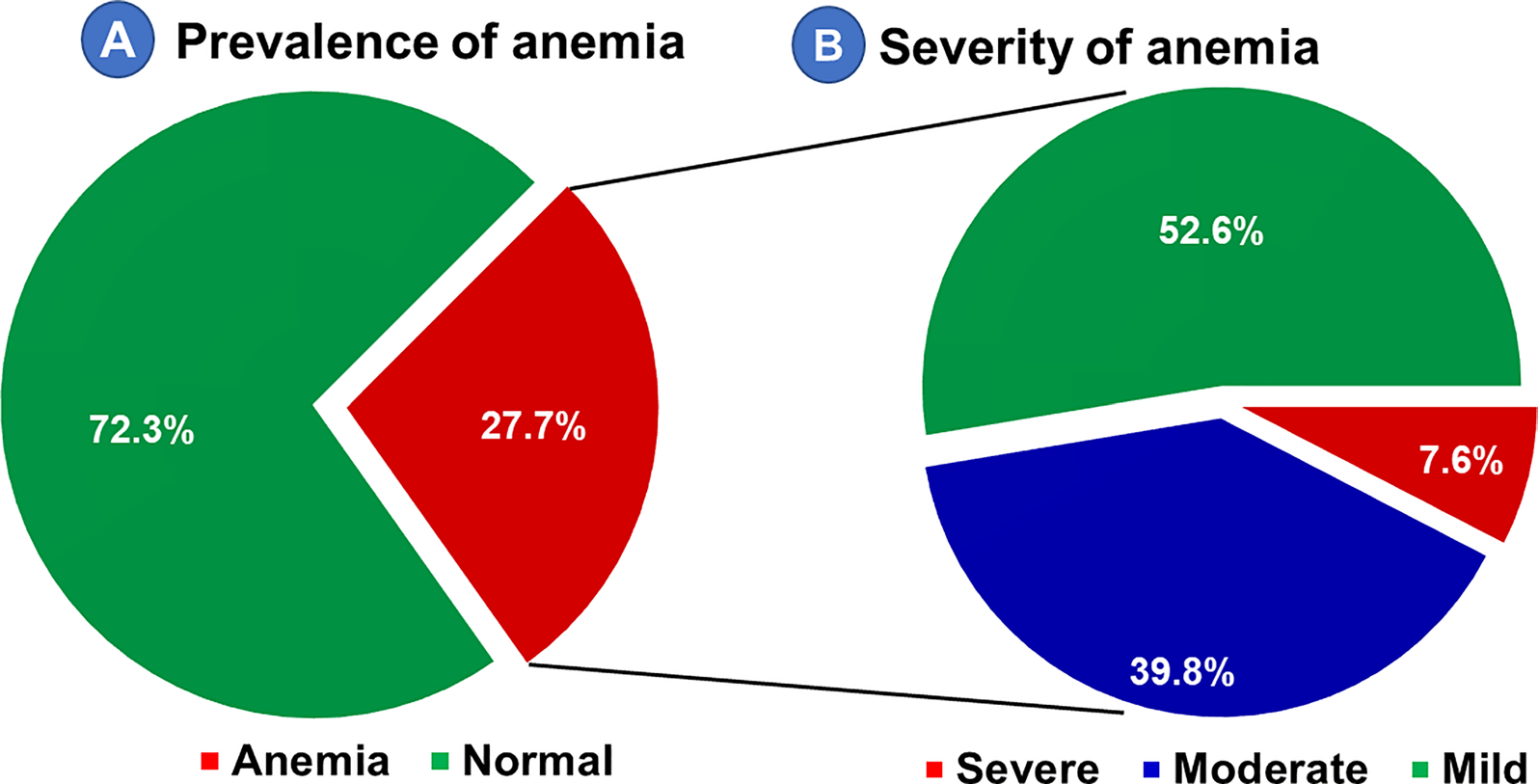
Prevalence (**A**) and severity (**B**) of anaemia among pregnant women in Majang Zone, Gambella, Southwest Ethiopia, November 2022–February 2023 [(n for prevalence = 425), (n for severity = 119)

The mean Hb concentration was 11.9 g/dl (95% CI 9.8, 14.2), with the lowest and highest of 5.4 g/dl and 20.1 g/dl, respectively. The Hb concentration decreased as parasite density increased (r = −0.58; P: 0.024) (Fig. 4). The prevalence of anaemia among pregnant women in the study area was 27.7% (95% CI 23.6, 32.1); above half (52.6%) of which was mild type (Fig. 5).

**Fig. 6:**
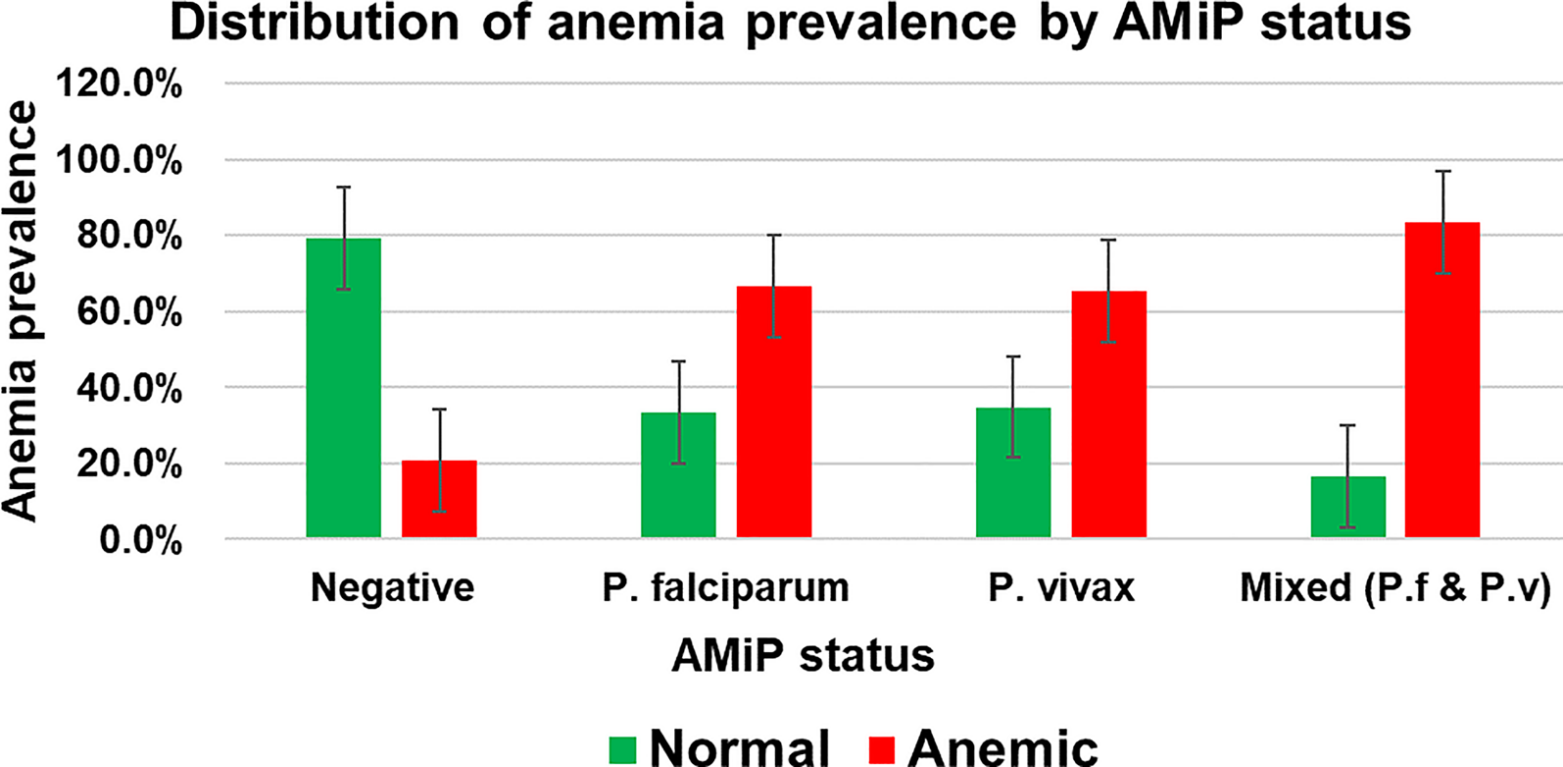
Distribution of anaemia by AMiP status among pregnant women in Majang Zone, Gambella, Southwest Ethiopia, November 2022–February 2023 (n = 425)

**Fig. 7:**
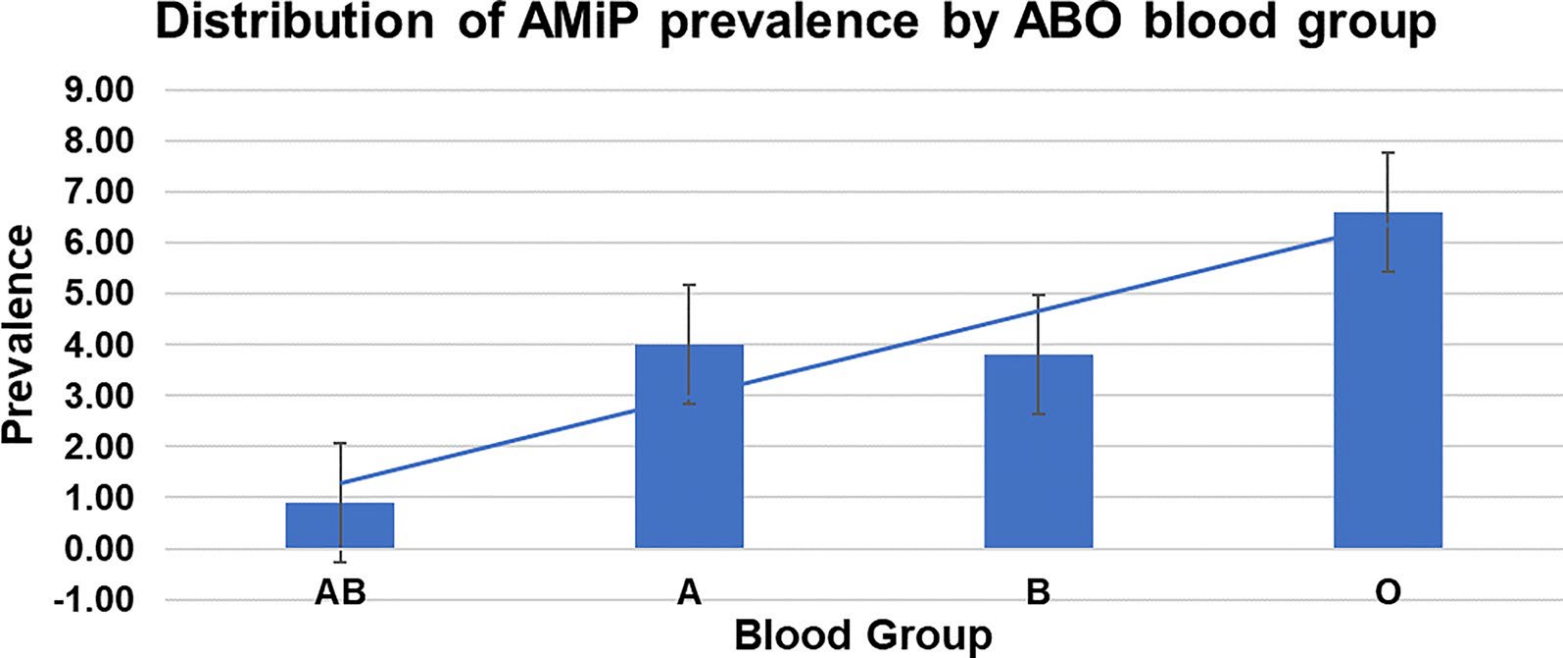
Distribution of AMiP by ABO blood group among pregnant women in Majang Zone, Gambella, Southwest Ethiopia, November 2022–February 2023 (n = 425)

The prevalence of anaemia among AMiP-positive pregnant women was greater (56.9%) than their counterparts (22.5%; P: 000). Pregnant women with AMiP had about fourfold increased odds of anaemia (AOR: 4.55 95% CI 2.63, 7.88). Over half of AMiP-positive pregnant women were anaemic, and *P. falciparum* was the major species that significantly increased the odds of anaemia (AOR: 5.41 95% CI 2.65, 11.06) (Fig. 6). The most and least common ABO blood groups in the study area were O (42.1%) and AB (7.3%), respectively. The prevalence of AMiP was higher among those with blood group O, and the difference was statistically significant (P: 0.026) (Fig. 7).

## Discussion

Asymptomatic malaria poses a hidden threat to malaria elimination efforts mainly because it escapes early detection and prompt management while serving as a source of infection that sustains transmission [2, 3, 59–62]. To ensure the effectiveness of malaria elimination efforts, it is critical to address segments of the general population with asymptomatic malaria, such as pregnant women, who have not received sufficient attention, particularly in Ethiopia, despite carrying high clinical and public health risks [3, 37, 42, 63]. Therefore, the current study aimed to assess the prevalence and risk factors of AMiP and anaemia in apparently healthy pregnant women attending ANC clinics at health facilities in Majang Zone of Gambella Region, Southwest Ethiopia.

The overall prevalence of AMiP among pregnant women attending ANC clinics at health facilities in Majang Zone was 15.3% (95% CI 12.0, 19.1). The prevalence of AMiP was 11.3%, 11.8%, and 17.6% by RDT, microscopy and qPCR, respectively. The distribution of *Plasmodium* species in the study area was *P. falciparum* (55.4%), *P. vivax* (35.4%) and mixed (9.2%). Moderate parasitaemia accounted for half (50.8%) of the overall infection's severity, with a geometric mean asexual parasitaemia of 2,683/ µl. The prevalence of submicroscopic parasitaemia was 3.6%.

This finding is consistent with the prevalence reported from similar facility-based cross-sectional studies done in Gurage Zone, Southern Ethiopia (15.2%) [32]; Jawi District, Northwest Ethiopia (18.1%) [35]; and Kwale County, Southern Kenya (12.9%) [25]. While *P. falciparum* was the dominant species in the study area, the overall distribution of *Plasmodium* species in the current study was *P. falciparum* (55.4%), *P. vivax* (35.4%) and mixed species (*P. falciparum* and *P. vivax*) (9.2%). This finding is nearly in line with the national malaria epidemiology [64] and the finding from a similar study done in Northwest Ethiopia [35]. The geometric mean asexual parasitaemia was 2,683/µl, while majority (50.8%) of *Plasmodium*-infected pregnant women carried moderate parasitaemia. This finding is concordant with the report from the study done in the rural surroundings of Arba Minch Town, South Ethiopia, where moderate parasitaemia dominated [34]. However, the parasite density was much higher than that was reported in Merti District, Central Ethiopia. This disparity might be due to the involvement of venous blood, community-based study design, and the timing of the study period relative to transmission season in their study [31].

The prevalence of AMiP in the present study was higher than the national pooled estimate of 7.8% reported by Tegegne et al*.* [65] and 7.2% reported by Duguma et al. [28]. These estimates were made based on studies, majority of which were community-based involving smaller sample size and conventional diagnostic tools [28, 65]. Likewise, this prevalence was higher than reports from Merti District (3.6%) [31], rural surroundings of Arba Minch Town (9.1% by microscopy and 9.7% by RDT) [34], and Boset District (2.74% by microscopy and 3.05% by RDT) [30]. The discrepancy might be due to methodological variation since their study period was short without covering high transmission season, community-based, smaller sample size, and did not use PCR assay unlike to the current study [7, 30, 31, 34]. Furthermore, the prevalence in the current study is higher than reports from Burkina Faso (11.0%) [24] and Bangladesh (3.2%) [23], both of which were community-based surveys covering larger geographical areas along different transmission seasons [23, 24].

On the other hand, the prevalence of AMiP in the current study was lower than the pooled 26.1% prevalence in sub-Saharan Africa [19], 36.4% in Tanzania [27], and 29.5% in The Democratic Republic of The Congo [26]. This discrepancy might be due to the larger sample size, longer study period, and inclusion of participants at their 1st ANC visit only in these studies [26, 27]. Furthermore, the prevalence found in this study is lower than the recent finding from West Guji Zone in Southern Ethiopia, where a 24.1% prevalence of AMiP was reported [33]. Inclusion of pregnant women with a history of malaria within the last month and participation of a large proportion of pregnant women who did not start ANC (64%) and lack ITN (72.3%) in that study might explain the disparity.

The present study identified four risk factors associated with AMiP: lack of ITN utilization within the previous week, having history of malaria within the previous year, lack of IRS spraying within the previous year and fewer frequency of ANC contact. Accordingly, these factors are discussed below.

The findings in the present study revealed that a lack of consistent and continued ITN utilization increased the risk of AMiP in the study area. Pregnant women who never slept under ITN within the previous week had over nine-fold (AOR: 9.43 95% CI 1.57, 56.62) increased odds of AMiP than those who used it every night. This finding is in agreement with reports from malaria-endemic regions in Colombia [22], Nassarawa-Eggon in Nigeria [66], Boset District in Central Ethiopia [30], and rural surroundings of Arba Minch Town in Southern Ethiopia [34]. The observed significant association could be explained by the fact that continued utilization of ITN reduces the risk of mosquito bites, thereby preventing *Plasmodium* infection [8, 42]. The recorded presence and absence of significant associations of ITN utilization and possession, respectively, with AMiP implies that having ITN by itself cannot guarantee protection against malaria, but consistently sleeping under it could reduce the lurking risk.

A history of malaria could increase the risk of AMiP, particularly in areas where *P. falciparum* and *P. vivax* are co-endemic through recurrence from either species or both [17]. Accordingly, pregnant women with a history of malaria within the previous year were by over twofold (AOR: 2.26 95% CI 1.16, 4.42) more likely to have an AMiP than their counterparts. This finding is consistent with the findings of previous studies that were undertaken in Merti District [31] and West Guji Zone [33] in Ethiopia. Given their ineligibility to radical cure anti-malarial drugs, such as PQ, it is physiologically and epidemiologically plausible that participants with a history of malaria within the previous year living in *P. falciparum* and *P. vivax* co-endemic area could have higher risk of relapse [1, 17, 59].

Another factor that was found to be significantly associated with AMiP was the status of IRS spraying in the pregnant woman’s house. Compared to those whose house was sprayed with IRS within the previous year, pregnant women living in a house that was not sprayed had a threefold (AOR: 3.00 95% CI 1.50, 6.00) higher odds of developing AMiP. This finding complements similar previous reports by Gemechu et al. [33] and Tilahun et al. [35], who also documented the presence of a statistically significant association between AMiP and IRS. It is entomologically plausible that spraying the house wall with IRS reduces the risk of malaria by killing or repelling mosquito mediating the transmission [45].

Pregnant women with only one ANC contact were about four times (AOR: 4.28 95% CI 2.06, 8.87) more likely to have AMiP as compared to those who had at least two rounds of ANC contacts. Studies conducted in Ethiopia [30] and Tanzania [27] revealed similar findings. In fact, it is epidemiologically and immunologically conceivable that pregnant women with a higher frequency of ANC contact could receive services such as advice, iron, and other healthcare services, including anti-malarials, that collectively improve pregnancy health and reduce the risk of infection, including malaria [30, 37].

The overall prevalence of anaemia among pregnant women was 27.7%. Although this prevalence is lower than that was reported in a study conducted in Pinyudo (36.1%) [67] in Gambella Region, it is categorized as a moderate public health problem [58]. The socioeconomic (refugee pregnant women including younger age), history of malaria, and slight geographic difference between the two studies might explain the observed discrepancy. In addition, pregnant women with AMiP had about fourfold increased odds of anaemia (AOR: 4.55 95% CI 2.63, 7.88). The prevalence of anaemia among malaria-positive pregnant women was greater than (56.9%) their counterparts (22.5%). Over half of malaria-positive pregnant women were anaemic, from which *P. falciparum* was the major species and found to be significantly increasing the odds of anaemia (AOR: 5.41 95% CI 2.65, 11.06). Consistent findings were reported from studies conducted in Kenya [25], Democratic Republic of Congo [26], and Tanzania [27]. Pregnant women with blood group O had a relatively higher prevalence of AMiP (43.1%). However, it did not show a statistically significant association (P > 0.05).

Generally, AMiP is a serious public health problem in the current study area. *Plasmodium*-infected pregnant women suffered from the high prevalence of anaemia showing the clinical burden, and a 4.9% prevalence of sexual parasitaemia reflecting the public health risk for transmission of malaria.

## Limitations of the study

The cross-sectional nature of the study design makes it difficult to establish a direct temporal relationship between AMiP and the associated risk factors. Due to lack of ultrasound in the facilities, the gestational age of some respondents was determined by LMP, which has limited accuracy. The gametocyte carriage rate was determined by microscopy, which has limited sensitivity compared to molecular tests rendering to potentially underestimate the prevalence.

## Conclusion and recommendation

This study showed that AMiP and anaemia are prevalent among pregnant women in the study area. *Plasmodium falciparum* is the most prevalent species in the area. Inadequate utilization of ITNs, lack of IRS spraying within the previous year, a history of malaria, and inadequate number of ANC contacts were the risk factors associated with AMiP. This study also showed that both RDT and microscopy missed a significant portion of infection, which tested positive with qPCR. Considering the dreadful impact of MiP and the ineligibility of pregnant women to many anti-malarial drugs while carrying a high prevalence, it is conceivable that AMiP is a daunting public health problem in the area. Pregnant women need strong attention to mitigate the burden of malaria and anaemia among them. Therefore, utilizing ITN more frequently, spraying with IRS, increasing ANC contact, and strengthening mitigation of MiP through use of more sensitive diagnostic tools could play significant roles in alleviating AMiP and anaemia in the current study area in particular and in Ethiopia in general.

## Data Availability

Data used for this research can be accessed from the corresponding author upon reasonable request.

## Abbreviations

ACT: Artemisinin-based combination therapy
AMiP: Asymptomatic malaria in pregnancy
BAb: Bound antibody
DNA: Deoxyribonucleic acid
DBSs: Dried blood spots
DHC: Dunchai health centre
FHC: Fejeji health centre
GMHC: Godere mission health centre
HRP: Histidine-rich protein
IPTp: Intermittent preventive treatment in pregnancy
IRS: Indoor residual spraying
ITN: Insecticide-treated net
KPH: Kumi primary hospital
LAb: Labelled antibody
LBW: Low-birth-weight
MHC: Metti health centre
qPCR: Quantitative polymerase chain reaction
RDT: Rapid diagnostic test
TIDRC: Tropical and infectious diseases research centre
